# Supplementary material for: The genome sequence of the Banded Ivy Tortrix moth, Clepsis dumicolana (Zeller, 1847)
Source: Wellcome Open Res. 2025 Mar 19;10:153. [Version 1] doi: 10.12688/wellcomeopenres.23905.1 (PMC12485335; doi:10.12688/wellcomeopenres.23905.1)
Supplement: Peer review history

# Reviewer report: Reviewer response for version 1

**DOI:** 10.21956/wellcomeopenres.26373.r121829

**Published:** 2025-05-07

**Authors:** Juan Galarza

**Affiliations:** 1 University of Oulu, Oulu, Finland

The genome sequence of the Banded Ivy Tortrix moth, Clepsis dumicolana (Zeller, 1847) by Lees et al., presents a good contribution to biodiversity genomics by producing a high-quality, chromosome-level genome assembly of *Clepsis dumicolana*, using well-established sequencing and assembly pipelines. The technical aspect is robust and the methods are described with enough detail to allow reproducibility. A strength of this genome note is the inclusion of a detailed biological background, which makes the paper useful beyond pure genomics. However, there are several points that need attention. First, the genome has not yet been annotated, and the manuscript should clearly explain the annotation plans. Second, while the authors mention depositing an alternate haplotype, they do not clarify the degree of phasing achieved or how the alternate data are organized relative to the primary assembly. Providing this information is essential for interpreting the dataset. Third, although quality metrics such as BUSCO scores and QV are impressive, in my opinion, the paper would be stronger with a brief comparison to related Lepidoptera genomes to give readers a sense of context. Overall, this is a valuable and well-executed genome note that would only need some clarifications and polishing.

Are sufficient details of methods and materials provided to allow replication by others?

Partly

Is the rationale for creating the dataset(s) clearly described?

Yes

Are the datasets clearly presented in a useable and accessible format?

Yes

Are the protocols appropriate and is the work technically sound?

Yes

Reviewer Expertise:

Molecular Ecology

I confirm that I have read this submission and believe that I have an appropriate level of expertise to confirm that it is of an acceptable scientific standard.

# Reviewer report: Reviewer response for version 1

**DOI:** 10.21956/wellcomeopenres.26373.r121310

**Published:** 2025-03-28

**Authors:** Kay Lucek

**Affiliations:** 1 University of Neuchâtel, Neuchâtel, Switzerland

The authors present the chromosome level genome assembly of a female specimen of the Banded Ivy Tortrix moth *Clepsis dumicolana*. The assembly consists of 30 chromosomes including both sex chromosomes but is not annotated for genes. The assembly is highly complete as revealed by the high BUSCO score but no information about phasing is given. Sequencing and genome assembly follow the current state of the art and use established methods. The assembly has been generated using the established pipelines of the Darwin Tree of Life Consortium.

 Overall, the presented assembly will be of great value to track the colonisation of this species in the UK and establish larger biogeographic studies. I appreciate the very detailed section on the biological background of the species as well as the methods section that is more extensive than other genome notes.

Are sufficient details of methods and materials provided to allow replication by others?

Yes

Is the rationale for creating the dataset(s) clearly described?

Yes

Are the datasets clearly presented in a useable and accessible format?

Yes

Are the protocols appropriate and is the work technically sound?

Yes

Reviewer Expertise:

Speciation genomics

I confirm that I have read this submission and believe that I have an appropriate level of expertise to confirm that it is of an acceptable scientific standard.
